# Role of Exercise Testing and Speckle Tracking Echocardiography in Paradoxical Severe Aortic Stenosis

**DOI:** 10.7759/cureus.18266

**Published:** 2021-09-25

**Authors:** Juan Lacalzada-Almeida, María Manuela Izquierdo-Gómez, Ignacio Laynez-Cerdeña, Amelia Duque-González, Leopoldo Pérez de Isla, Flor Baeza-Garzón, Alejandro Jiménez Sosa, Belén Marí-López

**Affiliations:** 1 Cardiology Department, Hospital Universitario de Canarias, Tenerife, ESP; 2 Cardiology Department, Hospital Clinico de San Carlos, Madrid, ESP; 3 Department of Research, Hospital Universitario de Canarias, Tenerife, ESP

**Keywords:** left ventricular strain, speckle tracking imaging, echocardiography, exercise testing, paradoxical aortic stenosis

## Abstract

Introduction

The clinical behavior and prognosis of patients with asymptomatic paradoxical low-gradient aortic stenosis (PLGAS) still remain controversial. Some authors consider PLGAS as an echocardiographically poorly quantified moderate AS (MAS). We aimed to investigate the clinical behavior of PLGAS by comparing it with that of asymptomatic high-gradient aortic stenosis (HG-AS) and MAS using transthoracic echocardiography (TTE) with speckle tracking imaging (STI) and cardiopulmonary exercise testing (CPET). The hypothesis of our study is, unlike that described by other authors, to demonstrate the existence of clinical and echocardiographic differences between PLGAS and MAS.

Methods

A cohort of 113 patients was included and categorized into three groups according to AS type: MAS (n=63), HG-AS (n=29), and PLGAS (n=21). Patients’ clinical data were obtained. Patients underwent 2D TTE with STI and CPET.

Results

There were no significant differences in the clinical variables between the three AS groups. In the multivariate multinomial logistic regression analysis, with PLGAS being the reference category, the most powerful variable for establishing a difference with HG-AS was the left ventricular mass (LVM) indexed by body-surface area (odds ratio [OR]=1.04, confidence interval (CI)=1.01-1.06, p<0.05). The MAS group showed less abnormal CPET (OR=0.198, CI=0.06-0.69, p<0.05), and higher left ventricle global longitudinal strain rate (GLSR) (OR=0.003, CI=0.00-0.35, p<0.05) than the PLGAS group.

Conclusions

TTE with STI and CPET established the clear differences between patients with asymptomatic PLGAS and those with asymptomatic MAS, as well as the similarities between patients with PLGAS and those with HG-AS. Our data identify PLGAS as a completely different entity from MAS.

## Introduction

Moderate valvular aortic stenosis (MAS), or severe aortic stenosis (AS), affects approximately 2.8% of the population aged >75 years [1]. The clinical behavior and prognosis of severe paradoxical low-gradient AS (PLGAS) still remain controversial. Some authors did not find any prognostic differences between patients with PLGAS and those with MAS [2-3], even going so far as to consider PLGAS as an echocardiographically poorly quantified MAS. Contrarily, other authors observed lower survival in patients with PLGAS than in those with high-gradient severe AS (HG-AS) [4-6].

Since the progression of AS is slow, and patients may have adapted a lifestyle to a poor functional status and falsely appearing asymptomatic, the evaluation with cardiopulmonary exercise testing (CPET) could provide objective information about the real functional capacity through the maximum consumption of O2, in addition to possible prognostic information to guide the proper clinical management of these patients [7-8]. On the other hand, echocardiographic myocardial deformation techniques can alert us to significant myocytic dysfunction before observing a deterioration of the left ventricular ejection fraction (LVEF) by the biplane method of disks (modified Simpson’s rule) with transthoracic echocardiography (TTE). Thus, numerous studies have reported a decrease in strain in severe AS patients compared to mild AS patients, which was related to a worse prognosis [9-12]. In addition, patients with symptomatic severe AS present a decrease in multidirectional strain compared to asymptomatic patients, even when having a preserved LVEF [13].

This study aimed to investigate the differences in the functional behavior and myocardial deformation parameters between patients with asymptomatic PLGAS and those with asymptomatic HG-AS and MAS using TTE with speckle tracking imaging (STI) and CPET. The hypothesis of our study is, unlike that described by other authors, to demonstrate the existence of clinical and echocardiographic differences between PLGAS and MAS [2-3].

## Materials and methods

Patient population and established groups

**Figure 1:**
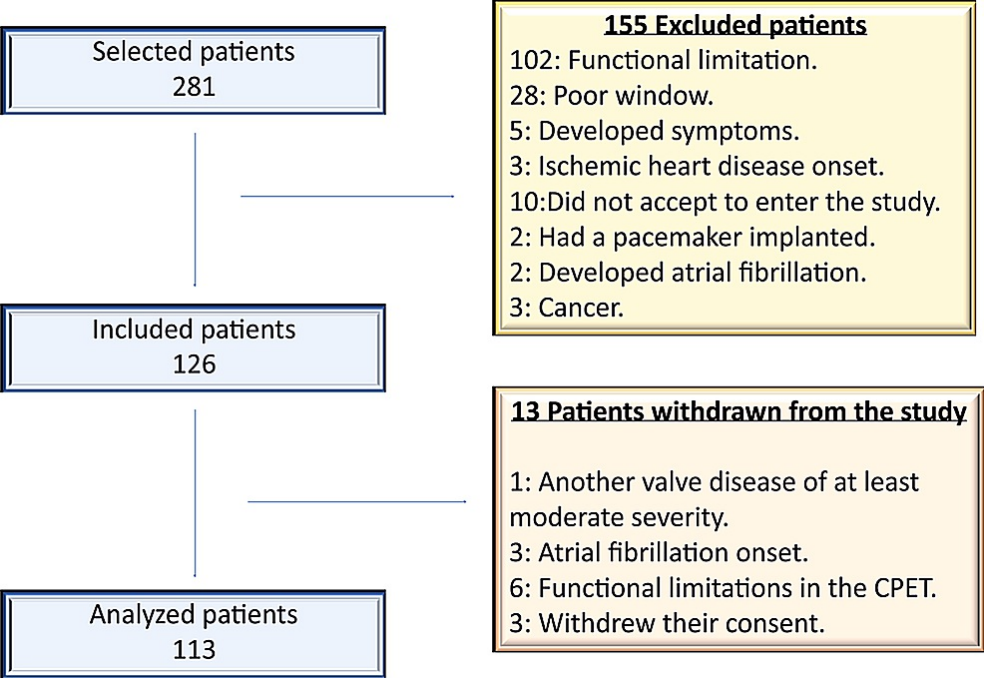
Flowchart of patients included and excluded in the study

For this single-center, prospective, observational study, a total of 281 consecutive patients with AS who presented at the cardiology department of University Hospital of Canary Islands from 2015 to 2018 were screened for inclusion. A cohort of 113 patients was included in the final analysis (Figure 1). The patients were categorized into the following three groups of AS: MAS, HG-AS, and PLGAS. The MAS was defined as AS with an aortic valve area (AVA) between 1-1.5 cm^2^ (0.6 - 0.85 cm^2^/m^2^), the HG-AS as AS with an AVA <1 cm^2^ (<0.6 cm^2^/m^2^), mean gradient (MG) >40 mmHg with a normal or diminished stroke volume index (SVi) and the PLGAS as AS with an AVA <1 cm^2^, MG <40 mmHg, SVi ≤ 35mL/m^2^ and LVEF ≥50% [7].

The inclusion criteria were as follows: patients aged >18 years; both sexes; asymptomatic; and diagnosed with MAS, HG-AS, or PLGAS with LVEF ≥60%. The exclusion criteria were the presence of a non-sinus rhythm, another significant valvulopathy, subvalvular or supravalvular AS, active endocarditis, known coronary disease (absence of known obstructive coronary artery disease, previous myocardial infarction, symptoms of angor pectoris or segmental left ventricle (LV) abnormalities of contractility on echocardiography), chronic obstructive pulmonary disease, malignant neoplasm under treatment, anemia, poor echocardiographic window, or physical inability to undergo CPET (Figure 1). A total of 128 patients were excluded. On the same day, TTE with STI and CPET was performed, TTE with STI first and CPET immediately after.

Transthoracic echocardiography

Conventional two-dimensional images with STI were acquired using a commercial ultrasound system applied to echocardiography (iE33 xMATRIX Koninklijke Philips NV, Eindhoven, The Netherlands) with a 2-4-​​MHz multifrequency probe. The analysis was performed offline by an expert observer using the Xcelera R2 echocardiographic analysis system, Philips Medical Systems (Amsterdam, Netherlands), following the recommendations of the American Society of Echocardiography [14].

Speckle tracking imaging

Second-harmonic images were obtained in B mode from the apical view (4, 2, and 3 chambers) and from the midventricular short axis. The images were acquired in grayscale with two-dimensional echocardiography with a sector narrowing of 30°-60° and an acquisition frequency of 60-90 images per second [15]. Subsequently and semi-automatically, the STI analysis was performed with appropriate software “QLAB Advance Tissue Motion Quantification v. 8.1 (Phillips)”. All measurements were performed offline by the same operator, who was blinded to the study protocol.

The global longitudinal strain (GLS) was calculated as the mean of the values ​​of the systolic peak of the longitudinal strain observed in each of the 16 segments of the left ventricle (LV). The global basal longitudinal strain of the LV was calculated as the mean of the values ​​of the systolic peak of the longitudinal strain of the ventricular basal segments. The mid-ventricular circumferential strain and the mid-ventricular radial strain systolic peak were determined as the mean of the values ​​of the six midventricular segments of the short axis [15].

Cardiopulmonary exercise testing

CPET was performed with a Marquette Case 8000 system (GE Medical, Chicago, Illinois) according to established guidelines [16-17], using the Bruce Rampa protocol [18]. All of the main classical variables of CPET were obtained. An abnormal CPET was defined if it ended prematurely due to dyspnea, chest pain, presyncope, or syncope. Other abnormality criteria were as follows: ST-segment depression of ≥2 mm measured at 80 ms from point J, ≥3 consecutive premature ventricular beats, and decreased or increased systolic blood pressure (SBP) by ≤20 mmHg from baseline. The parameters related to the gas analysis were not the criteria for discontinuing the CPET.

Statistical analyses

Continuous normally distributed data are presented as means with standard deviation (SD), which were compared between groups using student's t distribution. Variables that did not follow a normal distribution were expressed as the medians and interquartile range, which were compared between two groups using the Mann-Whitney U test. The categorical variables were expressed as absolute values with their corresponding percentages and were compared using the χ² test or Fisher's exact test. The Spearman’s rank test was used to assess the correlations between continuous variables. Continuous variables of the three AS groups were compared with the two-tailed analysis of variance (ANOVA) test and non-parametric variables were compared with the Kruskal-Wallis test. Univariate and multivariate multinomial logistic regression models were used with the backward step method; the three AS types were used as the dependent variables. The corresponding odds ratio (OR) and 95% confidence intervals (CI) for the different covariates were calculated. A univariate and multivariate linear regression analysis was performed with the entire sample in order to verify the strength in the relationship between the types of AS and the significant independent variables previously found in the univariate analysis. The intra and interobserver variabilities for strain measurements were analyzed using the Bland-Altman test.

Statistical analysis was performed with SPSS version 21 software (IBM Corp., Armonk, NY). The two-tailed p-value of <0.05 was considered significant.

## Results

Study population

The patients’ mean age was 74 SD, 8 years, and the study cohort was predominantly male (54%). There were no significant differences in the clinical and demographic variables among the three AS types, except for SBP (p = 0.001) and pulse pressure (p = 0.001), which showed higher values in the HG-AS group than in the other two groups (Table 1 and Appendix 1).

**Table 1:** Clinical and echocardiographic variables of patients according to the classification of aortic stenosis SD: standard deviation; AS: aortic stenosis; MAS: moderate aortic stenosis; HG-AS: high-gradient severe aortic stenosis; PLGAS: paradoxical low-gradient aortic stenosis; SBP: systolic blood pressure; DBP: diastolic blood pressure; CPET: cardiopulmonary exercise testing. IEV: indexed ejection volume; PG: peak gradient; LVEF: left ventricular ejection fraction; AVA: aortic valve area; LVM: left ventricular mass; ZVA: valvuloarterial impedance; GLS: global longitudinal strain; GLSR: global longitudinal strain rate; LS: longitudinal strain; ANOVA: analysis of variance

Variables	MAS (a) (n: 63)	HG-AS (b) (n: 29)	PLGAS (c) (n: 21)	p ANOVA	p Post-Hoc
Age (years)	74 (70 to 80)	76 (72 to 81)	74 (67 to 78)	0.475	
Gender (male)	50.8%	62.1%	52.4%	0.594	
SBP (mmHg)	130 (120 to 140)	140 (130 to 140)	130 (120 to 130)	0.001	a vs. b = 0.001; b vs. c = 0.001
DBP (mmHg)	70 (70 to 80)	80 (70 to 80)	70 (70 to 80)	0.126	
Pulse pressure	60 (50 to 60)	60 (50 to 70)	50 (50 to 60)	0.001	a vs. b < 0.05; b vs. c = 0.001
Abnormal CPET	20 (31.7%)	16 (55.2%)	14 (66.7%)	<0.05	a vs. b < 0.05; a vs. c < 0.05
Dyspnea in CPET	14 (22.2%)	8 (27.6%)	9 (42.9%)	0.186	
ST Decline ≥ 2 mm	5 (7.9%)	10 (34.5%)	4 (19%)	<0.05	a vs. b = 0.001
CPET basal SBP (mmHg)	120 (110 to 130)	140 (130 to 145)	125 (110 to 130)	0.001	a vs. b < 0.05; b vs. c = 0.001
CPET max. SBP (mmHg)	150 (140 to 150)	155 (150 to 160)	130 (120 to 140)	<0.05	a vs. b = 0.001; b vs. c < 0.05
PG (mmHg)	39.65 (32.8 to 44.35)	101.5 (83.2 to 104.4)	43.05 (42.8 to 43.3)	<0.001	a vs. b < 0,001; a vs. c = 0,05; b vs. c < 0,001
LVEF (%)	72.5 (67 to 76.1)	75.4 (70 to 79.3)	68.05 (64 to 72.1)	0.102	
Indexed AVA (cm^2^/m^2^)	0.69 SD 0.8	0.41 SD 0.08	0.45 SD 0.08	<0.001	a vs. b < 0.001; a vs. c < 0.001; b vs. c < 0.001
Indexed LVM (g/m^2^)	99.28 SD 22.69	132.32 SD 35.16	106.01 SD 25.92	<0.001	a vs. b < 0.001; b vs. c < 0.05
Z_VA (_mmHg/ml/m^2^)	3.4 SD 0.70	4.12 SD 0.72	4.26 SD 1.07	<0.001	a vs. b < 0.001; a vs. c < 0.001
LV GLS (%)	-14.37 SD 2.65	-13.72 SD 1.94	-12.65 SD 1.80	< 0.05	a vs. c < 0.05
LV GLSR (1/s)	-0.77 (-0.86 to -0.65)	-0.62 (-0.72 to -0.55)	-0.60 (-0.68 to -0.54)	< 0.05	a vs. b < 0.05; a vs. c = 0.001
Global Basal LS (%)	-15.37 SD 3.10	-13.48 SD 3.42	-13.44 SD 3.49	< 0.05	a vs. b < 0.05; a vs. c < 0.05
Basal GLSR (1/s)	-0.95 (-1.13 to -0.80)	-0.86 (-0.95 to -0.70)	-0.92 (-0.99 to -0.73)	< 0.05	a vs. b < 0.05

The most frequent cause of initial exclusion was the inability to perform CPET (36.3 %), followed by a poor echocardiographic window (9.9%). The other reasons are found in Figure 1. From the 113 patients analyzed, 63 presented MAS (55.8%), 29 presented HG-AS (25.7%), and 21 presented PLGAS (18.6%).

Results of CPET according to the AS classification

In total, 44.2% of the patients had abnormal CPET. According to the AS types, 66.7%, 55.2%, and 31.7% of the PLGAS, HG-AS, and MAS groups, respectively, had abnormal CPET, with the differences being significant (p<0.05) (Table 1 and Appendix 6).

Significant differences were found in baseline SBP during CPET (p = 0.001), maximum SBP during CPET (p <0.05), and decrease in ST-segment ≥2 mm (p <0.005) (Table 1 and Appendix 6). All of the 19 patients with a decrease in ST-segment underwent coronary angiography and only one had coronary artery disease. Post-hoc analysis revealed that patients with MAS had a lower proportion of abnormal CPET than patients with HG-AS and PLGAS (p<0.05). Patients with HG-AS had CPET basal SBP, and CPET SBP during maximum effort was greater than in those with MAS and PLGAS (p <0.05 and 0.001, respectively). There were no significant differences in the CPET variables (Appendix 1).

Result of echocardiographic variables according to AS classification

In the post-hoc analysis, we observed that the HG-AS group had greater left ventricular mass (LVM) indexed by body surface area (BSA) than the MAS and PLGAS groups (p <0.001 and p <0.05, respectively) (Table 1 and Appendix 7 (panel A)).

The relative wall thickness (RWT) was >0.42 in all groups, but it was more prominent in the HG-AS group than in the MAS and PLGAS groups. This finding was consistent with the increase in the left atrium, with the HG-AS group showing a higher increase compared to the other groups (Appendix 2 and Appendix 7 (panel B)).

The MAS patients had a lower valvuloarterial impedance (ZVA) than the HG-AS (p <0.001) and PLGAS (p <0.001) patients (Table 1 and Appendix 7 (panel C)).

Results of echocardiographic variables of myocardial deformation according to AS classification

The HG-AS group had a worse LV global longitudinal strain rate (GLSR) than the MAS group [-0.62 (-0.72 to -0.55) vs. -0.77 (-0.86 to -0.65), respectively (p <0.05)]. The PLGAS group also had a worse GLSR than the MAS group [-0.60 (-0.68 to -0.54) vs. -0.77 (-0.86 to -0.65), respectively (p = 0.001)]. The HG-AS and PLGAS groups had a worst global basal longitudinal strain than the MAS group (both p <0.05). The GLS of the LV was worse in the PLGAS group than in the MAS group (-12.65 SD 1.8 vs. -14.37 SD 2.65, respectively, p <0.05) (Table 1).

We did not observe a difference in the distribution of the circumferential and radial deformities of the LV among the three groups (Appendix 2).

Bland-Altman analysis showed good intra- and inter-observer agreement with a non-significant bias. The intraobserver and interobserver variabilities for GLS were 1.18% (95% CI, 1.09%-1.31%) and 1.31% (95% CI, 1.15%-1.82%), respectively.

Univariate multinomial logistic regression analysis

Among the differences observed between PLGAS and MAS, the following data were prominent in PLGAS: higher number of abnormal CPET (p <0.05), worst GLS (p <0.05), worst GLSR (p <0.05), and worst global basal longitudinal strain (p <0.05) (Table 2 and Appendix 3).

**Table 2:** Univariate and multivariate multinomial analysis results Reference category: PLGAS: paradoxical low-gradient aortic stenosis. MAS: moderate aortic stenosis; HG-AS: high-gradient severe aortic stenosis; OR: odds ratio; CI: confidence interval; CPET: cardiopulmonary exercise testing; LVM: left ventricular mass; ZVA: valvuloarterial impedance; GLS: global longitudinal strain; GLSR: global longitudinal strain rate

Univariate analysis						
Variables	MAS			HG-AS		
	OR	95% CI	p	OR	95% CI	p
Abnormal CPET	0.23	0.08-0.67	< 0.05	0.62	0.19-1.97	0.410
Indexed LVM (g/m^2^)	0.99	0.97-1.01	0.260	1.04	1.01-1.06	< 0.05
LV GLS (%)	0.71	0.55–0.91	< 0.05	0.8	0.61-1.04	0.096
LV GLSR (1/s)	0.001	0.00-0.10	< 0.05	0.03	0.00-2.78	0.127
Multivariate analysis						
AS Grade	Variables	p	OR	CI		
HG-AS	Indexed LVM	< 0.05	1.04	1.01-1.06		
	Abnormal CPET	0.46	0.61	1.17-2.25		
	LV GLSR	0.13	0.02	0.00-2.99		
MAS	Indexed LVM	0.57	0.99	0.97-1.02		
	Z_VA_	0.001	0.262	0.12-0.59		
	Abnormal ST	< 0.05	0.198	0.06-0.69		
	LV GLSR	< 0.05	0.003	0.00-0.35		

When comparing the PLGAS and HG-AS groups, the PLGAS group had lower SBP (p = 0.001), lower pulse pressure (p = 0.001), lower basal SBP during CPET (p = 0.001), lower maximum SBP during CPET (p <0.05), lower LVM indexed by BSA (p <0.05), and lower left atrial diameter (p = <0.05) (Table 2 and Appendix 3).

Multivariate multinomial logistic regression analysis

When comparing MAS with the reference category (PLGAS), MAS showed lesser abnormal CPET (OR = 0.198 CI = 0.06-0.69, p <0.05) and better GLSR of LV (OR = 0.003 CI = 0.00-0.35, p <0.05); these variables characterized the difference between the two groups (Table 2). The variable ZVA was not used in this analysis to avoid collinearity with the dependent variable.

With PLGAS used as the reference category, the most powerful variable in establishing a difference with respect to HG-AS was the LVM indexed by BSA (OR = 1.04 CI = 1.01-1.06, p <0.05), with HG-AS showing a greater indexed LVM than PLGAS (Table 2).

Unadjusted and adjusted standardized linear regression coefficients

Linear regression analyzes were performed with the dependent variables indexed being LVM and LV GLSR. These two variables had shown an excellent correlation with the type of AS in the logistic regression analysis (Table 2). In this way, the total sample of patients studied was used, not being fragmented according to the three types of AS. The indexed LVM showed correlation, both in the unadjusted and adjusted models, with the type of AS and the LV GLSR (Appendix 4).

With the linear model that included the LV GLSR as a dependent variable, they showed correlation, both in the unadjusted model and in the adjusted one, with the type variables of AS and the METS in the CPET (Appendix 5).

## Discussion

To the best of our knowledge, this study is the first to comparatively analyze the functional behavior of asymptomatic patients with MAS, PLGAS, and HG-AS using TTE with STI and CPET. Our findings demonstrated that PLGAS is a completely different form of AS from MAS and that, on the other hand, PLGAS has similar behavior to HG-AS.

Jander et al. [2] and Tribouilloy et al. [3] found that the prognosis of PLGAS is similar to that of MAS, although other authors indicated that PLGAS has a lower survival than HG-AS [4-6]. Although there is no consensus about the prognosis of patients with PLGAS, a meta-analysis published in 2016 concluded that these patients presented a prognosis similar to those with HG-AS [19].

There are no comparative data on the behavior in CPET between PLGAS and MAS or HG-AS, to characterize them as authentic severe AS or not. In our study, we observed a clear difference in abnormal CPET among the AS groups. However, no difference was found in the CPET gas analysis, which could be probably due to the careful patient selection, wherein symptomatic patients or with doubtful clinical data were excluded. Regarding ZVA in PLGAS and MAS, a study established that a ZVA of ≥4.5 mmHg/ml/m^2^ would indicate lower survival [20]. Patients with PLGAS usually have higher ZVA related to the greater myocardial dysfunction, possibly indicating a more advanced stage of AS [4,21]. In our series, we observed greater ZVA in the PLGAS group than in the MAS group. This finding could not be attributed to the greater SBP because there were no differences in the SBP between the two groups, being directly related to the aortic valve area.

Our study resembles that of Maréchaux et al. who observed how patients with MAS, with a lower value of ZVA, had a higher value of GLS and GLSR as compared with patients with HG-AS and PLGAS, whose ZVA value was higher [22]. However, despite these differences, no significant differences were observed in LVEF. Despite what was published about its prognosis [2-3], we considered that ZVA, GLS, and GLSR of the LV are essential parameters to differentiate PLGAS and MAS, however, the variable ZVA was not used in the regression analysis.

Regarding the deformation of the LV, the GLS and GLSR were significantly decreased in the PLGAS group as compared with the MAS group. Even in the multivariate analysis, this difference in the GLSR was maintained. Recently, it has been reported that deterioration of GLS in PLGAS would be a predictor of poor prognosis, suggesting a benefit in asymptomatic patients with early aortic valve replacement [23]. We have demonstrated this significant decrease in the longitudinal deformity despite the absence of difference in LVEF between the two groups. Thus, the systolic deformity of the LV is a more robust marker of myocardial dysfunction than LVEF determined with the biplane method of disks (modified Simpson’s rule), which allowed the differentiation between PLGAS and MAS. In addition, the ease of determining LV systolic deformity compared to a high percentage of patients in our sample where CPET could not be performed.

In our series, when we analyzed the behavior of PLGAS against HG-AS, PLGAS has a behavior similar to HG-AS. The variable that best differentiated PLGAS from HGAS was LVM indexed by BSA, being significantly increased in the latter. Conversely, Hachicha et al. observed greater concentric remodeling in PLGAS than in HG-AS [4]. This, in our series, would be justified by the significant difference found between PLGAS and HG-AS in SBP, with higher values presented in HG-AS, which would indicate concentric remodeling of the LV by AS and hypertension.

Both groups, PLGAS and HG-AS, presented decreased GLS compared with the normal published values (-19.7% to -22.4%) [15,24-25]. Donal et al. described similar results of GLS in patients with asymptomatic severe AS, which was greatly reduced compared to those of healthy controls [26]. Other authors observed that PLGAS with decreased GLS had a prognosis similar to HG-AS, but, if GLS was normal, the prognosis resembled that of the normal flow-low‐gradient severe AS group, whose behavior is compared with the MAS group [27], concluding that GLS marked the prognosis of the PLGAS group. CPET did not show significant differences between the PLGAS and HG-AS groups.

Our results support the theory that PLGAS is an entity similar to HG-AS since both present similar results in CPET and have decreased SGL but without significant differences.

Strengths and limitations

First, the exclusion criteria of our study, especially due to functional limitations of the patients when performing CPET, mean that the data obtained are not fully extrapolated to the entire population with AS. However, they portray the real life of patients with AS at an advanced age.

Second, the sample size of patients with PLGAS may have been insufficiently large to detect other relevant significant differences, but it was enough to show that PLGAS is a different entity from MAS.

Third, the presence of asymptomatic coronary artery disease could alter the results of CPET and strain values; however, all patients with a history of coronary heart disease were carefully excluded.

Fourth, our results of myocardial deformation by STI should be interpreted with caution when compared with those from other authors who have used different software programs. In addition, the absence of healthy controls to compare these results may be a limitation.

## Conclusions

In our study, PLGAS is a different entity from MAS, presenting worse results in CPET and greater deterioration of GLSR. However, PLGAS resembled HG-AS, showing similar results in CPET and GLS. Therefore, we considered that the first comparison characterizes two differentiated entities; therefore, their clinical management should also be as such. As for the second comparison, there would be two similar entities that could benefit from similar management.

The combined use of CPET and TTE with STI allows for a better characterization of the functional behavior of the asymptomatic patient with PLGAS and its differentiation from the patients with MAS.

## Figures and Tables

**Figure 2:**
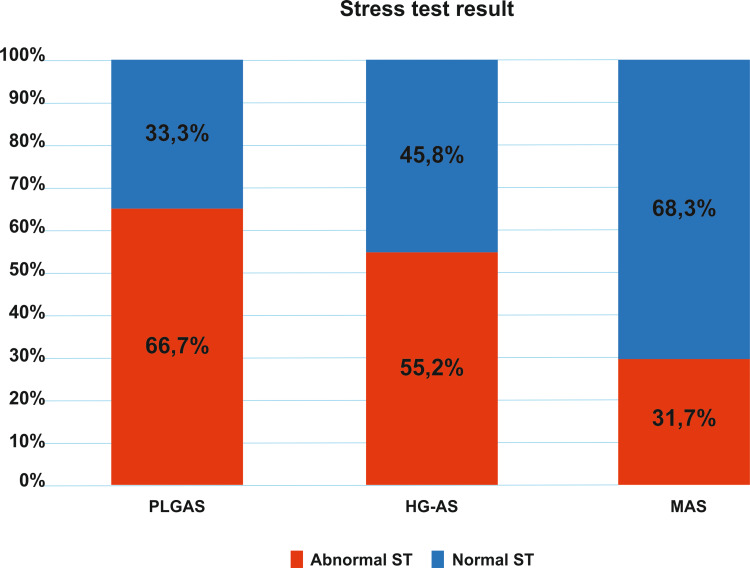
Results of the cardiopulmonary exercise testing according to the aortic stenosis classification

**Figure 3:**
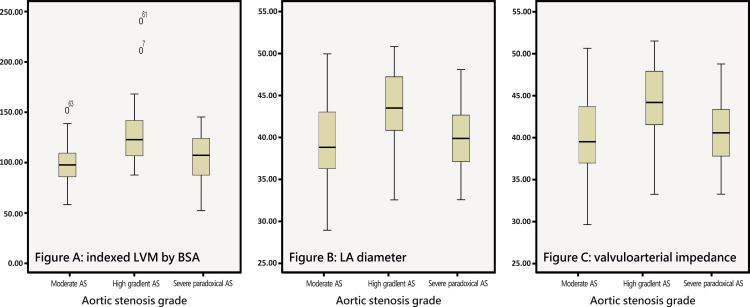
According to the classification of the aortic stenosis distribution of the indexed LVM (A), size of LA (B), and valvuloarterial impedance (C) LVM indicates left ventricular mass, BSA indicates body-surface area, LA indicates left atrium

## Appendices

Appendix 1 

**Table 3:** Patient characteristics according to the AS classification AS: aortic stenosis; MAS: moderate aortic stenosis; HG-AS: high-gradient severe aortic stenosis; PLGAS: paradoxical low-gradient aortic stenosis; SD: standard deviation; BSA: body surface area; CPET: cardiopulmonary exercise testing; HR: heart rate; DBP: diastolic blood pressure; METs: metabolic equivalents; Max VO2.: maximum oxygen consumption; VO2/Kg max.: maximum oxygen consumption per kilogram of weight; VE: ventilation; VCO2: carbon dioxide production; RER: respiratory ratio

Variables	MAS (a) (n: 63)	HG-AS (b) (n: 49)	PLGAS (c) (n: 41)	P	Post-Hoc P
Arterial hypertension	55 (87.3%)	42 (86.2%)	33 (81%)	0.768	
Diabetes mellitus	26 (41.9%)	17 (34.5%)	22 (52.4%)	0.449	
Dyslipidemia	45 (71.4%)	44 (89.7%)	29 (71.4%)	0.085	
Smoker/ex-smoker	18 (28.5%)	19 (37.9%)	18 (42.8)	0.516	
BSA, m^2^	1.82 SD 0.19	1.83 SD 0.17	1.86 SD 0.2	0.644	
Ventricular arrhythmias	2 (3.2%)	7 (13.8%)	4 (9.5%)	0.162	
Effort angina	0 (0%)	2 (3.4%)	0 (0%)	0.232	
CPET basal HR, bpm	68 (58 to 82)	70 (61 to 83)	51 (40 to 62)	0.309	
CPET maximum HR, bpm	129 SD 18	126 SD 17	127 SD 24	0.728	
CPET basal DBP, mmHg	70 (65 to 75)	80 (70 to 80)	65 (60 to 70)	0.261	
CPET maximum DBP, mmHg	80 (75 to 80)	80 (80 to 80)	70 (60 to 80)	0.238	
CPET BP drop, mmHg	2 (3.2%)	5 (10.3%)	2 (4.8%)	0.360	
METs	5.08 SD 2.55	5.53 SD 2.62	4.77 SD 2.16	0.551	
Max. VO_2, _mL. min^-1^	1503 SD 440.31	1585.55 SD 474.32	1520.05 SD 576.40	0.743	
Max. VO_2 _/Kg, mL. Kg-1^. ^min^-1^	20.05 SD 4.86	20.48 SD 4.9	19.1 SD 6.10	0.636	
Max. VO_2_. % compared to expected	100 (98 to 100)	98 (91 to 100)	84 (69 to 100)	0.264	
VE/VCO_2 _Slope (VCO_2_/VO_2_)	26 (24 to 27)	27 (25 to 28)	26.5 (24 to 29)	0.408	
RER<1.1	22 (34.9%)	12 (24.1%)	8 (19%)	0.301	

Appendix 2 

**Table 4:** Echocardiographic variables and myocardial deformation according to the AS classification AS: aortic stenosis; MAS: moderate aortic stenosis; HG-AS: high-gradient severe aortic stenosis; PLGAS: paradoxical low-gradient aortic stenosis; SVi: stroke volume index; Vmax: maximum velocity; Vmed: medium velocity; MG: medium gradient; AVA: aortic valvular area; IVS: interventricular septum; LVPW: left ventricular posterior wall; RWT: relative wall thickness; LA: left atrium; LVTDD: left ventricular telediastolic diameter; LVTDV: left ventricular telediastolic volume; BSA: body surface area; LVTSD: left ventricular telesystolic diameter; LVTSV: left ventricular telesystolic volume; DT: deceleration time; MCS: midventricular circumferential strain; MCSR: midventricular circumferential strain rate; MRS: midventricular radial strain; MRSR: midventricular radial strain rate

Variables	MAS (a) (n: 63)	HG-AS (b) (n:49)	PLGAS (c) (n:41)	ANOVA P	Post-Hoc P
SVi, ml/m^2^	45.06 SD 4.33	41.61 SD 4.85	29.32 SD 4.6	<0.001	a vs. b < 0.001 b vs. c < 0.001
Vmax., m/s	314.35 (285.7 to 333)	503.6 (456.1 to 509.3)	327.6 (327 to 328.2)	<0.001	a vs. b < 0.001 a vs. c < 0.05 b vs. c < 0.001
Vmed., m/s	200 (175.6 to 228.75)	349.3 (307.5 to 375.7)	216.5 (210.4 to 222.6)	<0.001	a vs. b < 0.001 a vs. c < 0.05 b vs. c < 0.001
MG, mmHg	19.45 (15.1 to 24.6)	57.4 (43.4 to 62.60)	21.8 (20.6 to 23)	<0.001	a vs. b < 0.001 a vs. c < 0.05 b vs. c < 0.001
AVA, cm^2^	1.2 (1.1 to 1.3)	0.69 (0.55 to 0.84)	0.9 (0.89 to 0.91)	<0.001	a vs. b < 0.001 a vs. c < 0.001
IVS thickness, mm	11.75 SD 2.12	13.48 SD 2.18	11.75 SD 1.92	0.001	a vs. b = 0.001 b vs. c < 0.05
LVPW thickness, mm	10.89 SD 1.86	12.59 SD 1.78	10.82 SD 1.98	<0.001	a vs. b < 0.001 b vs. c < 0.05
RWT	0.52 SD 0.11	0.57 SD 0.11	0.49 SD 0.09	<0.05	b vs. c < 0.05
LA Size, mm	40.40 SD 5.54	44.86 SD 5.01	40.95 SD 4.38	0.001	a vs. b = 0.001 b vs. c < 0.05
Aortic root, mm	29.18 SD 4.16	30 SD 4.1	29.85 SD 4.6	0.625	
E/A Ratio	0.77 (0.67 to 1.05)	0.72 (0.68 to 0.99)	1.1 (0.9 to 1.3)	0.266	
LVTDD, mm	44.24 SD 5.01	46.90 SD 6.11	46.71 SD 4.84	<0.05	
LVTDV/BSA,ml/m^2^	42.29 SD 9.9	45.45 SD 16.13	43.99 SD 16.3	0.114	
LVTSD, mm	27 (24 to 30)	28 (24 to 30)	32.5 (27 to 38)	0.431	
LVTSV/BSA, ml/m^2^	11.47 (8.08 to 13.45)	10.79 (8.17 to 13.74)	18.3 (12.7 to 23.89)	0.733	
Transmitral DT, ms	265.87 SD 69.88	288.62 SD 101.37	242.45 SD 75.31	0.140	
MCS, %	-14.01 SD 4.41	-15.73 SD 2.62	-12.84 SD 4.07	0.147	
MCSR, 1/s	-0.8 (-1 to -0.6)	-0.9 (-1.1 to -0.8)	-0.52 (-0.53 to -0.5)	0.082	
MRS, %	20.75 SD 13.19	19.86 SD 12.37	22.08 SD 14.12	0.901	
MRSR, 1/s	1.57 SD 0.85	1.43 SD 0.69	1.35 SD 0.81	0.665	

Appendix 3 

**Table 5:** Results of the univariate multinomial analysis The reference category is: PLGAS. MAS: moderate aortic stenosis; HG-AS: severe high gradient aortic stenosis; PLGAS: paradoxical low-gradient aortic stenosis; OR: odds ratio; CI: confidence interval; SBP: systolic blood pressure; CPET: cardiopulmonary exercise testing; SVi: stroke volume index; Vmax: maximum velocity; PG: peak gradient; Vmedia: medium velocity; MG: medium gradient; LVM: left ventricular mass; LA: left atrium; LVTDD: left ventricular telediastolic diameter; SL: longitudinal strain; LSR: longitudinal strain rate

Variables	MAS			HG-AS		
	OR	95% CI	p	OR	95% CI	p
SBP, mmHg	1.02	0.99-1.06	0.240	1.09	1.036-1.147	0.001
Pulse Pressure, mmHg	1.05	0.993-1.11	0.083	1.13	1.05-1.21	0.001
CPET basal SBP, mmHg	1.03	0.99-1.07	0.174	1.1	1.04-1.15	< 0.001
CPET max. SBP, mmHg	1.01	0.97-1.05	0.650	1.06	1.01-1.12	< 0.05
SVi, mL/m^2^	1.29	1.13-1.47	< 0.001	1.32	1.14-1.52	< 0.001
Vmax., m/s	0.98	0.96-0.99	< 0.05	1.12	1.03-1.22	< 0.05
PG, mmHg	0.92	0.87-0.97	< 0.05	1.19	1.08-1.32	< 0.001
Vmed., m/s	0.99	0.97-1	< 0.05	1.21	1.05-1.39	< 0.05
MG, mmHg	0.87	0.79-0.95	< 0.05	1.93	1.2-3.11	< 0.05
LVM, g	0.99	0.98-1.00	0.140	1.02	1.003-1.03	< 0.05
LA diameter, mm	0.98	0.89-1.08	0.680	1.17	1.04-1.32	< 0.05
LVTDD, mm	0.92	0.83-1.08	0.071	1.01	0.90-1.12	0.902
Global Basal SL, %	0.829	0.71-0.97	< 0.05	0.997	0.84-1.18	0.969
Global Basal LSR, 1/s	0.157	0.02-1.25	0.080	1.079	0.11-10.73	0.950

Appendix 4 

**Table 6:** Unadjusted and adjusted standardized linear regression coefficients, with dependent variable indexed LVM AS: aortic stenosis. *The reference category is paradoxical low-gradient aortic stenosis. SBP: systolic blood pressure; CPET: cardiopulmonary exercise testing; METs: metabolic equivalents; Max. VO2/Kg: maximum oxygen consumption per kilogram of weight; VE: ventilation; VCO2: carbon dioxide production; RER: respiratory ratio; LV: left ventricular; GLS: global longitudinal strain; GLSR: global longitudinal strain rate; GLS: global longitudinal strain; GLSR: global longitudinal strain rate

	Unadjusted simple regression		Adjusted multiple regression^*^	
Variable	b Coefficient Standardized	P	b Coefficient Standardized	P
AS type*	0.447	< 0.001	0.273	0.002
Age	0.081	0.392		
Gender	0.242	0.01		
Arterial hypertension	0.078	0.410		
Diabetes mellitus	0.056	0.558		
SBP, mmHg	0.231	0.014		
Abnormal CPET	0.077	0.415		
Dyspnea in CPET	-0.071	0.453		
ST decline ≥ 2 mm	0.124	0.190		
METs	0.174	0.065		
Max. VO_2 _/Kg, mL^. ^Kg-1^. ^min^-1^	0.155	0.101		
VE/VCO_2 _Slope	0.089	0.350		
RER (VCO_2_/VO_2_)	0.122	0.200		
LV GLS, %	0.061	0.524		
LV GLSR, 1/s	0.381	< 0.001	0.214	0.026
GLS Basal LV, %	0.344	< 0.001		
GLSR Basal, 1/s	0.049	0.613		

Appendix 5 

**Table 7:** Unadjusted and adjusted standardized linear regression coefficients, with dependent variable left ventricle global longitudinal strain. AS: aortic stenosis. *The reference category is paradoxical low-gradient aortic stenosis. SBP: systolic blood pressure; CPET: cardiopulmonary exercise testing; METs: metabolic equivalents; Max. VO2/Kg: maximum oxygen consumption per kilogram of weight; VE: ventilation; VCO2: carbon dioxide production; RER: respiratory ratio

	Unadjusted simple regression		Adjusted multiple regression^*^	
Variable	b Coefficient Standardized	p	b Coefficient Standardized	p
AS type*	0.228	0.015	0.197	0.027
Age	0.109	0.251		
Gender	-0.133	0.895		
Arterial hypertension	0.195	0.039		
Diabetes mellitus	0.135	0.155		
SBP, mmHg	0.039	0.684		
Abnormal CPET	-0.010	0.916		
Dyspnea in CPET	0.075	0.431		
ST decline ≥ 2 mm	-0.123	0.193		
METs	-0.269	0.004	-0.280	0.002
Max. VO_2 _/Kg, mL. Kg-1^. ^min^-1^	-0.258	0.006		
VE/VCO_2 _Slope	0.145	0.126		
RER (VCO_2_/VO_2_)	-0.217	0.021		

Appendix 6 

Appendix 7 
